# What changed between the peak and plateau periods of the first COVID-19 pandemic wave? A multicentric Portuguese cohort study in intensive care

**DOI:** 10.5935/0103-507X.20210037-en

**Published:** 2022

**Authors:** Rui Antunes Pereira, Marta Sousa, José Pedro Cidade, Luís Melo, Diogo Lopes, Sara Ventura, Irene Aragão, Raul Miguel de Freitas Lima Neto, Elena Molinos, Ana Marques, Nelson Cardoso, Flávio Marino, Filipa Brás Monteiro, Ana Pinho Oliveira, Rogério C Silva, André Miguel Neto Real, Bruno Sarmento Banheiro, Renato Reis, Maria Adão-Serrano, Ana Cracium, Ana Valadas, João Miguel Ribeiro, Pedro Póvoa, Camila Tapadinhas, Vítor Mendes, Luís Coelho, Raquel Maia, Paulo Telles Freitas, Isabel Amorim Ferreira, Tiago Ramires, Luís Silva Val-Flores, Mariana Cascão, Rita Alves, Simão C Rodeia, Cleide Barrigoto, Rosa Cardiga, Maria João Ferreira da Silva, Bruno Vale, Tatiana Fonseca, Ana Lúcia Rios, João Camões, Danay Pérez, Susana Cabral, Maria Inês Ribeiro, João João Mendes, João Gouveia, Susana Mendes Fernandes

**Affiliations:** 1 Hospital de Curry Cabral, Centro Hospitalar Universitário Lisboa Central - Lisboa, Portugal; 2 Hospital de Santa Maria, Centro Hospitalar Universitário Lisboa Norte - Lisboa, Portugal; 3 Hospital São Francisco Xavier, Centro Hospitalar Universitário Lisboa Ocidental - Lisboa, Portugal; 4 Hospital Professor Doutor Fernando Fonseca - Amadora, Portugal; 5 Hospital de São José, Centro Hospitalar Universitário Lisboa Central - Lisboa, Portugal; 6 Hospital de Santo António, Centro Hospitalar do Porto - Porto, Portugal; 7 Hospital Vila Nova de Gaia-Espinho - Vila Nova de Gaia, Portugal; 8 Hospital Pedro Hispano, Unidade Local de Saúde de Matosinhos - Matosinhos, Portugal; 9 Centro Hospitalar Universitário de Coimbra - Coimbra, Portugal; 10 Hospital Espírito Santo de Évora - Évora, Portugal; 11 Hospital de Vila Franca de Xira - Vila Franca de Xira, Portugal; 12 Hospital de Egas Moniz, Centro Hospitalar Universitário Lisboa Oriental - Lisboa, Portugal; 13 Centro Hospitalar de Tondela-Viseu - Tondela e Viseu, Portugal; 14 Hospital de Santa Luzia, Unidade Local de Saúde Alto Minho - Viana do Castelo, Portugal; 15 Hospital de Abrantes, Centro Hospitalar do Médio Tejo - Abrantes, Portugal; 16 Hospital de Portimão, Centro Hospitalar Universitário do Algarve - Portimão, Portugal; 17 Sociedade Portuguesa de Cuidados Intensivos - Lisboa, Portugal

**Keywords:** COVID-19, Coronavirus infections, SARS-CoV-2, Pandemics, Intensive care, Critical illness, Adrenal cortex hormones, Acute respiratory distress syndrome, Critical care outcomes

## Abstract

**Objective:**

To analyze and compare COVID-19 patient characteristics, clinical management and outcomes between the peak and plateau periods of the first pandemic wave in Portugal.

**Methods:**

This was a multicentric ambispective cohort study including consecutive severe COVID-19 patients between March and August 2020 from 16 Portuguese intensive care units. The peak and plateau periods, respectively, weeks 10 - 16 and 17 - 34, were defined.

**Results:**

Five hundred forty-one adult patients with a median age of 65 [57 - 74] years, mostly male (71.2%), were included. There were no significant differences in median age (p = 0.3), Simplified Acute Physiology Score II (40 *versus* 39; p = 0.8), partial arterial oxygen pressure/fraction of inspired oxygen ratio (139 *versus* 136; p = 0.6), antibiotic therapy (57% *versus* 64%; p = 0.2) at admission, or 28-day mortality (24.4% *versus* 22.8%; p = 0.7) between the peak and plateau periods. During the peak period, patients had fewer comorbidities (1 [0 - 3] *versus* 2 [0 - 5]; p = 0.002) and presented a higher use of vasopressors (47% *versus* 36%; p < 0.001) and invasive mechanical ventilation (58.1 *versus* 49.2%; p < 0.001) at admission, prone positioning (45% *versus* 36%; p = 0.04), and hydroxychloroquine (59% *versus* 10%; p < 0.001) and lopinavir/ritonavir (41% *versus* 10%; p < 0.001) prescriptions. However, a greater use of high-flow nasal cannulas (5% *versus* 16%, p < 0.001) on admission, remdesivir (0.3% *versus* 15%; p < 0.001) and corticosteroid (29% *versus* 52%, p < 0.001) therapy, and a shorter ICU length of stay (12 days *versus* 8, p < 0.001) were observed during the plateau.

**Conclusion:**

There were significant changes in patient comorbidities, intensive care unit therapies and length of stay between the peak and plateau periods of the first COVID-19 wave.

## INTRODUCTION

The surge of the coronavirus disease 2019 (COVID-19) pandemic represented a tremendous challenge for health care systems worldwide, particularly in intensive care units (ICUs). Six months after the COVID-19 pandemic declaration on the 11th of March 2020, over 28 million cases of severe acute respiratory syndrome coronavirus 2 (SARS-CoV-2) infection and 917,000 deaths had been reported.^(1)^ Furthermore, it has been estimated that approximately 26% of hospitalized COVID-19 patients required ICU admission.^(2)^ Worldwide reports of mortality rates among critical patients varied widely, ranging from 26% to 97%.^(2-10)^

In Portugal, during the first six months of the SARS-CoV-2 pandemic between March and August, the total number of confirmed infections in the country reached 58,012, with an overall mortality rate of 3.1%. In the first wave, the peak of confirmed community infections was reached on the 26th of March 2020 and was linked with increased health care system stress and risk of ICU bed shortage, a consequence of a low number of ICU beds (6.4/100000 habitants).^(11)^ A national lockdown cancelled nonemergent clinical activity and increased ICU bed available for critically ill COVID-19 patients.

Early COVID-19 clinical practice and guidelines were changed as data emerged during the initial phases of the pandemic. As a result, epidemiologic data comparing distinct temporal periods of the first pandemic wave are scarce.^(3,4,6-8,10)^ Clinical data on severely ill COVID-19 patients in the ICU are crucial for improved care, in-hospital patient flow and health care system organization.

This study aimed to analyze and compare COVID-19 patient characteristics, clinical management and outcomes between the peak and plateau periods of the first pandemic wave in Portugal.

## METHODS

We performed a multicentric ambispective observational cohort study open to all ICUs between the 1st of March and the 31st of August 2020 in Portugal. The study was endorsed by the *Sociedade Portuguesa de Cuidados Intensivos*. The ISARIC (International Severe Acute Respiratory and emerging Infections Consortium) was a key partner and source of the standardized clinical data collection tool used by each participating center before the final database merger for this study.^(12)^

Patients with a primary diagnosis of SARS-CoV-2 polymerase chain reaction (PCR)-confirmed pneumonia admitted to intensive care units between the 1st of March and 31st of August 2020 were eligible for this study. Patients were consecutively included and followed-up until hospital discharge.

All patients without complete hospital stays by the end of the study period and SARS-CoV-2-infected patients admitted to the ICU for other reasons were excluded from the analysis.

Study variables were collected from the clinical records and included demographics, clinical data, comorbidities, signs and symptoms, laboratory results, therapeutics, length of stay (LOS) and mortality. These variables were collected at hospital admission, ICU admission and hospital discharge. Missing, illogical and outlier values were reported to local investigators for correction, and the final database resulted from the combination of the datasets from each center collected independently.

The initial peak and the following plateau periods corresponded to weeks 10 - 16 and 17 - 34 of 2020. These periods were defined by histogram analysis of the frequency of patient admission in the ICUs during the first wave of the SARS-CoV-2 pandemic, revealing two clear periods with peak and plateau characteristics, corresponding to a cutoff value of 20 new patient admissions per week.

### Statistical analysis

Categorical variables were described as counts and percentages. Dichotomic variables were compared using the chi-square test or Fisher’s exact test as appropriate. For comparisons between groups, the Kruskal-Wallis nonparametric test was used to test whether multiple categories within each variable originated from the same distribution.

Continuous variables were described as the mean and standard deviation (SD) or median and interquartile range (IQR) as appropriate, comparisons were made using t tests or ANOVA for parametric variables, and Mann-Whitney tests were used for nonparametric variables.

Multivariate analysis was performed using logistic regression to assess whether age, sex and comorbidities predicted mortality, as described in a Portuguese population-based cohort study, after adjusting for severity of illness using the Simplified Acute Physiology Score II (SAPS II) score.^(13)^

Statistical analysis was performed using IBM Statistical Package for the Social Science (SPSS) for Windows, version 23.0 and RStudio Team.

This study was approved by the National Ethics Committee for Clinical Research (2020_EO_02) and the Ethics Committees of each center. Informed consent was waived given the observational character of this study and the exceptional context of the COVID-19 pandemic. This study complied with the ethical principles of the Declaration of Helsinki. The Strengthening the Reporting of Observational Studies in Epidemiology (STROBE) Statement guidelines for reporting observational studies were used for this manuscript.

## RESULTS

### Participating centers and patients

**Figure 1:**
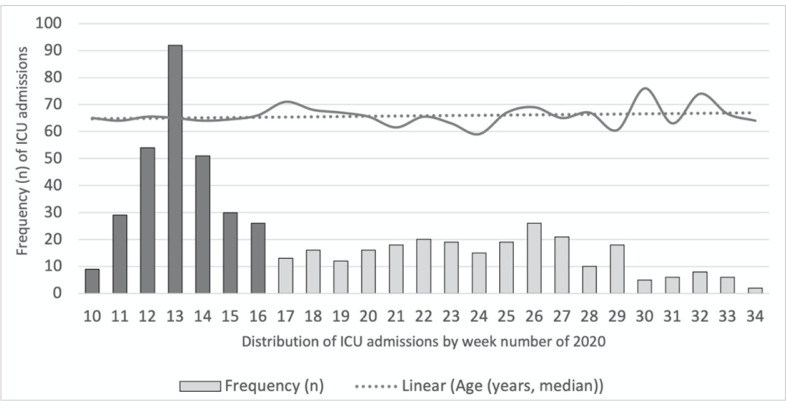
Intensive care unit admissions and median age of COVID-19 patients.

Sixteen centers provided data on 596 adult critical COVID-19 patients for the study (Table 1S - Supplementary material). Seven SARS-CoV-2-positive patients were excluded because the primary diagnosis for ICU admission was not pneumonia but acute coronary syndrome or pyelonephritis. Additionally, forty-eight patients from the plateau period were excluded due to continued hospitalization at the time of database closure. The main analysis across the 6-month period included 541 adult patients (Figure 1). Pediatric and neonatal ICUs from four centers provided clinical data for seven children that were separately described (Table 2S - Supplementary material).

The peak period occurred during weeks 10-16: n = 291, median age 65 [58 - 71] years and 28-day mortality 24.4%. The plateau period occurred during weeks 17 - 34: n = 250, median age 66 [57 -76], 28-day mortality 22.8%. There was no significant difference in age or mortality rate score between the peak and plateau periods (p > 0.05).

ICU - intensive care unit.

### Epidemiology

Adult patients in this study were mostly male (71.2%) with a median age of 65 [57 - 74] years, and arterial hypertension (47.1%) was the most frequent comorbidity. Approximately one-third (32.7%) of patients had no comorbidities reported, and these were younger than the others (63 [54 - 68] years *versus* 67 [59 - 76], p < 0.001). Patient demographic characteristics and comorbidities are detailed in table 1 and hospital presenting symptoms are presented in table 3S (Supplementary material).

**Table 1:** Baseline characteristics of critical COVID-19 patients and comparison between peak and plateau periods of the first wave in 2020

	Overall	Peak (weeks 10 - 16)	Plateau (weeks 17 - 34)	p value
	541	291	250	
Age (years)	65 [57 - 74]	65 [58 - 71]	66 [57 - 76]	0.3
Male sex	385 (71.2)	206 (70.8)	179 (71.6)	0.9
Number of comorbidities	2 [0 - 4]	1 [0 - 3]	2 [0 - 5]	0.002
Hypertension	255 (47.1)	126 (43.3)	129 (51.6)	0.07
Obesity	150 (27.7)	78 (26.8)	72 (28.8)	0.7
Cardiovascular disease	81 (15.0)	37 (12.7)	44 (17.6)	0.1
Pulmonary disease^*^	65 (12.0)	27 (9.3)	38 (15.2)	0.048
Renal disease	55 (10.2)	25 (8.6)	30 (12.0)	0.2
Neurologic disease†	26 (4.8)	12 (4.1)	14 (5.6)	0.5
Neoplasm disease	26 (4.8)	13 (4.5)	13 (5.2)	0.8
Liver disease	22 (4.1)	12 (4.1)	10 (4.0)	1.0
Asthma	15 (2.8)	10 (3.4)	5 (2.0)	0.5
Hematologic disease	15 (2.8)	6 (2.1)	9 (3.6)	0.4
Diabetes mellitus	15 (2.8)	15 (5.2)	0	
Dementia	8 (1.5)	1 (0.3)	7 (2.8)	0.045
HIV/AIDS	7 (1.3)	3 (1.0)	4 (1.6)	0.8
GCS	15 [15 - 15]	15 [14 - 15]	15 [15 -15]	0.04
Hemoglobin (mg/dL)	12.6 [11.2 - 13.9]	12.6 [11.2 - 14.1]	12.7 [11.2 - 13.9]	0.7
White blood cell (10^9/mL)	8.3 [5.6 - 11.2]	8.3 [5.7 - 11.5]	8.1 [5.5 - 10.9]	0.5
Platelet (10^9/mL)	207 [154 - 280]	213 [166 - 287]	204 [150 - 279]	0.11
Total bilirubin (mg/dL)	0.5 [0.4 - 0.8]	0.6 [0.4 - 0.9]	0.5 [0.4 - 0.8]	0.048
Creatinine (mg/dL)	1.0 [0.7 - 1.4]	1.0 [0.7 - 1.3]	1.0 [0.7 - 1.4]	0.9
C-reactive protein (mg/dL)	158 [100 - 242]	155 [102 - 240]	162 [98 - 244]	0.9
PaO2/FiO2 ratio	138 [101 - 202]	139 [101 - 209]	136 [101 - 195]	0.6
pH	7.42 [7.34 - 7.46]	7.42 [7.35 - 7.47]	7.41 [7.33 - 7.46]	0.2
Lactate (mmol/L)	1.4 [1.0 - 8.0]	1.20 [1.0 - 3.0]	1.8 [1.1 - 9.0]	0.001
SAPS II (n = 527)	40 [31 - 52]	40 [31 - 52]	39 [30 - 51]	0.8
IMV‡ (n = 464)	292 (54.0)	169 (58.1)	123 (49.2)	< 0.001
HFNC‡ (n = 404)	55 (10.2)	14 (4.8)	41 (16.4)	< 0.001
NIV‡ n = 405)	34 (6.3)	14 (4.8)	20 (8.0)	0.3
ECMO‡ (n = 406)	6 (1.1)	3 (1.0)	3 (1.2)	0.9
Vasopressors‡ (n = 464)	226 (41.8)	137 (47.1)	89 (35.6)	< 0.001
RRT‡ (n = 464)	20 (3.7)	6 (2.1)	14 (5.6)	< 0.001
Antibiotics§ (n = 311)	187 (60.1)	99 (57.2)	88 (63.8)	0.2
Onset of symptoms to hospital (days)	6[4 - 9]	7 [4 - 9]	6 [3 - 8]	0.002
Hospital to ICU admission (days)	1 [0 - 3]	1 [0 - 3]	1 [0 - 3]	0.7

GCS - Glasgow coma score; PaO_2_ - partial arterial oxygen pressure; FiO_2_ - fraction of inspired oxygen; SAPS II - Simplified Acute Physiology Score II; IMV - invasive mechanical ventilation; HFNC - high flow nasal cannula; NIV - noninvasive ventilation; ECMO - extracorporeal membrane oxygenation; RRT - renal replacement therapy; ICU - intensive care unit.

* Nonasthma pulmonary disease. † nondementia neurologic disease. ‡ therapies used during the intensive care unit admission day. § antibiotic therapy initiated 24 hours before or after intensive care unit admission. The frequency (n) is indicated whenever it differs from the overall (n = 541). Results expressed as n (%) or median [interquartile range].

### Clinical severity, management and mortality

At ICU admission, the Simplified Acute Physiology Score (SAPS) II (n = 527) presented a median value of 40 [31 - 52]. The types of respiratory support provided during ICU admission are detailed in table 1. Antibiotic prescription data (n = 311) showed that in 60.1% (n = 187) of cases, prescription took place at admission (24 hours before or after ICU admission), and azithromycin alone or in combination was present in 70.0% of these.

Throughout the ICU stay, nearly two-thirds (61.7%) of the patients were reported to have severe acute respiratory distress syndrome (ARDS); respiratory support, therapies in the ICU, and outcomes are shown in table 2.

**Table 2:** COVID-19 acute respiratory distress syndrome severity, therapies and clinical results during the intensive care unit stay and comparison between peak and plateau periods of the first wave in 2020

	Overall	Peak (weeks 10 - 16)	Plateau (weeks 17 - 34)	p value
	541	291	250	
ARDS (n = 334)				0.2
Mild	13 (2.4)	5 (1.7)	8 (3.2)	
Moderate	107 (19.8)	63 (21.6)	44 (17.6)	
Severe	214 (39.6)	122 (41.9)	92 (36.8)	
IMV (n = 520)	399 (73.8)	238 (81.8)	161 (64.4)	< 0.001
ECMO (n = 414)	24 (4.4)	11 (3.8)	13 (5.2)	0.4
Vasopressors (n = 409)	296 (54.7)	175 (60.1)	121 (48.4)	< 0.001
RRT (n = 474)	91 (16.8)	47 (16.2)	44 (17.6)	< 0.001
Prone positioning (n = 408)	221 (40.9)	130 (44.7)	91 (36.4)	0.04
Antibiotics (n = 403)^*^	323 (80.1)	185 (85.3)	138 (74.2)	0.006
Antivirals (n = 403)	275 (50.8)	201 (69.1)	74 (29.6)	< 0.001
Hidroxichloriquine	197 (36.4)	172 (59.1)	25 (10.0)	< 0.001
Lopinavir/ritonavir	144 (26.6)	119 (40.9)	25 (10.0)	< 0.001
Remdesivir	38 (7.0)	1 (0.3)	37 (14.8)	< 0.001
Antifungals (n = 400)	39 (7.2)	17 (5.8)	22 (8.8)	0.3
Corticosteroids (n = 403)	216 (39.9)	85 (29.2)	131 (52.4)	< 0.001
Tracheostomy (n = 414)	42 (7.8)	22 (7.6)	20 (8.0)	0.5
Survival at Day 28	413 (76.3)	220 (75.6)	193 (77.2)	0.7
ICU survival	412 (76.2)	215 (73.9)	197 (78.8)	0.2
Hospital survival	390 (72.1)	205 (70.4)	185 (74.0)	0.4
ICU LOS (days)	10 [5 - 19]	12 [5 - 22]	8 [4 - 16]	0.001
Hospital LOS (days)	22 [13 - 37]	23 [14 - 41]	21 [12 - 33]	0.02

ARDS - acute respiratory distress syndrome; IMV - invasive mechanical ventilation; ECMO - extracorporeal membrane oxygenation; RRT - renal replacement therapy; ICU - intensive care unit; LOS - length-of-stay.

* Antibiotics prescribed throughout the ICU stay. Kruskal-Wallis nonparametric test test was used to test whether categories within each variable originated from the same distribution. Results expressed as n (%) or median [interquartile range].

Overall, the 28-day mortality rate was 23.7%, in-ICU 23.8% and in-hospital 27.9% (Table 2). Patients receiving invasive mechanical ventilation (IMV) (73.8%) during their ICU stay presented a 28-day mortality rate comparable to those receiving any other type of noninvasive oxygen support (respectively, 25.3% *versus* 17.4%, p = 0.09). There were no reports of patients receiving IMV outside the ICU settings.

**Figure 2:**
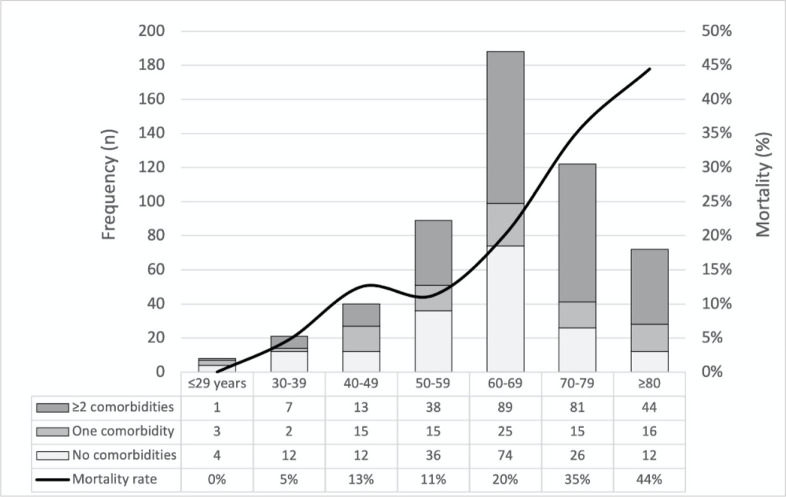
Age group, comorbidities and associated intensive care unit mortality rates in critically ill COVID-19 patients.

Age groups, comorbidities and associated ICU mortality rates are depicted in figure 2.

Mortality risk factor analysis (n = 526) revealed that older age (adjusted odds ratio -aOR 1.05; confidence interval - 95%CI 1.03 - 1.07; p < 0.001) was independently associated with increased ICU mortality after adjustment for SAPS II score (aOR 1.02; 95%CI 1.01 - 1.04; p = 0.002), while the number of comorbidities (aOR 1.09; 95%CI 0.90 - 1.06; p = 0.5) and male sex (aOR 0.8; 95%CI 0.50 - 1.24; p = 0.3) were not.

### Peak and plateau phase of the first COVID-19 pandemic wave

The temporal distribution of ICU admissions, age and mortality rate between the peak and plateau periods are depicted in figure 1. The peak period occurred between weeks 10 and 16, with an abrupt increase in the number of ICU admissions to a maximum of 92 during week 13, followed by a plateau period between weeks 17 and 34. Approximately half of the patients (53.8%) included in this study were admitted to the ICU during the peak period.

The baseline characteristics of COVID-19 patients between the peak and plateau of the first SARS-CoV-2 wave are detailed in table 1. The number of days from the onset of symptoms until hospital admission (7 [4 - 9] *versus* 6 [3 - 8]; p = 0.002) or until ICU admission (9 [6 - 11 *versus* 7 [5 - 10], p = 0.003) were higher during the peak than in the plateau period, and no differences were found regarding the time between hospital to ICU admission, age or severity of illness, as assessed by SAPS II score between periods (Table 1).

During the peak period, patients presented fewer comorbidities (1 [0 - 3] *versus* 2 [0 - 5]; p = 0.002) and displayed significantly more vasopressor use (47.1% *versus* 35.6%; p < 0.001) and a higher frequency of IMV (58.1 *versus* 49.2%; p < 0.001) at ICU admission. Conversely, in the plateau period, there was an increase in high-flow nasal cannula (HFNC) use (4.8% *versus* 16.4%; p < 0.001) at ICU admission, although there were no significant differences in the partial arterial oxygen pressure/fraction of inspired oxygen ratio (PaO_2_/FiO_2_; 139 *versus* 136; p = 0.6) between periods (Table 2).

Significant therapeutic differences between the peak and plateau periods were observed, with a reduction in hydroxychloroquine (59.1% *versus* 10.0%; p < 0.001) and lopinavir/ritonavir (40.9% *versus* 10.0%; p < 0.001) and an increase in remdesivir (0.3% *versus* 14.8%; p < 0.001) and corticosteroid therapy (29.2% *versus* 52.4%, p < 0.001). There was no significant difference in the proportion of antibiotics prescribed 24 hours before or after ICU admission (Table 1), although throughout the entire ICU stay, there was a reduction in overall antibiotic prescription between peak and plateau periods (Table 2). Finally, there was a significant decrease in median ICU LOS (days) (12 [5 - 22] *versus* 8 [4 - 16]; p < 0.001) and hospital LOS (23 [14 - 41] *versus* 21 [12 - 33]; p = 0.02) and no significant difference in 28-day mortality (24.4% *versus* 22.8%; p = 0.7) between peak and plateau periods (Table 2).

## DISCUSSION

In this study, we showed that clinical characteristics and management of patients admitted to the ICU during the peak and plateau periods of the first COVID-19 pandemic wave in Portugal were different despite similar age, severity of illness and 28-day mortality rate. During the peak period, patients presented fewer comorbidities and had a higher use of IMV, vasopressors, prone positioning, and hydroxychloroquine and lopinavir/ritonavir administration. The plateau period was characterized by higher rates of use of HFNC for respiratory support, increased prescription of remdesivir and corticosteroid therapy, and shorter hospital and ICU LOS.

Although the majority of hospitalized patients and overall confirmed SARS-CoV-2 infections in Portugal were female (59% and 55%, respectively), in this cohort of critically ill COVID-19 patients, there was a preponderance of men, which is in line with other studies reporting up to 60-80% of patients in this setting as male.^(13-18)^ Gender-specific immune responses could provide a possible explanation for these findings.^(19)^

A high proportion of patients in our study presented comorbidities, although the number of comorbidities was not associated with 28-day mortality.^(13,15,20)^ Of note, we observed patients with more comorbidities in the plateau phase, suggesting an admission bias toward more fit patients in the peak phase. We speculate that this may have been a consequence of less strict criteria for ICU admission, resulting from a larger availability of beds following the lockdown period and the reduction in the ICU admission rate in the plateau period, but our data do not draw such conclusions.

There was a higher frequency of IMV use at ICU admission during the peak period, although clinical severity (SAPS II and PaO_2_/FiO_2_ ratio) at ICU admission was similar in both periods. These differences could result from the delay between the onset of symptoms until the first hospital encounter in the emergency department, leading to the need for urgent decisions to “intubate and ventilate” by impending severe respiratory failure due to COVID-19 during the peak period. Furthermore, initial COVID-19 recommendations considered that HFNC and noninvasive ventilation (NIV) could be detrimental for hypoxemic patients and increased viral shedding with a potentially higher risk for health care professionals, leading to patient intubation and ventilation in emergency departments and wards for safer ICU transfer.^(21)^ As safety data emerged, these recommendations were updated to include NIV and HFNC in the clinical management of hypoxemic patients and postponed the “intubate and ventilate” decision in the later plateau period of the pandemic.

Major differences regarding off-label compassionate use of COVID-19 therapies including three repurposed drugs (hydroxychloroquine, lopinavir/ritonavir and remdesivir) and corticosteroids were observed between periods, in parallel with new data.^(22)^ The use of hydroxychloroquine and lopinavir/ritonavir in the treatment of COVID-19 was initially suggested due to their in vitro inhibition of coronavirus SARS infection.^(23,24)^ These drugs did not show any clinical benefit in randomized clinical trials (RCTs) and raised concerns for adverse reactions, such as gastrointestinal disorders and cardiotoxicity, with prolongation of the corrected QT interval, particularly in the case of hydroxychloroquine coadministered with azithromycin.^(23,25-27)^ Remdesivir inhibited SARS-CoV-2 replication in human epithelial cells, and double-blind placebo-controlled RCTs reported a reduction in time to clinical improvement in COVID-19 hospitalized patients as well as a significant reduction in 28-day mortality in patients requiring oxygen support.^(28-32)^ However, the larger SOLIDARITY open label RCT did not show any clinical benefit for hydroxychloroquine, lopinavir/ritonavir or remdesivir in either ventilated or nonventilated patients. Currently, these COVID-19 compassionate use therapies are not formally recommended in the treatment of critically ill patients.^(33,34)^

Antibiotic therapy was consistently prescribed at ICU admission throughout our study. This reflected concerns of bacterial coinfection; however, its incidence in ICU COVID-19 patients has been reported to be low (8.1 - 14%).^(35-37)^ Additionally, the immunomodulatory properties of azithromycin have shown no clinical benefit, and the routine use of antibiotics in COVID-19 patients is not supported by evidence.^(38)^

Our study presented a large proportion of COVID-19 patients treated with corticosteroids, with a significant increase during the plateau phase. This increase coincided with preliminary results of the RECOVERY trial, available after 16 June 2020, showing a significant reduction in 28-day mortality in hospitalized patients who were receiving either IMV or oxygen alone and were treated with dexamethasone.^(39)^ These findings were later corroborated by the CoDEX trial.^(40)^ The increase in corticosteroid therapy between periods in our cohort shows how swiftly clinical practice changed to incorporate data available from these RCTs.

Finally, dynamic changes in the community, national policies, health care systems and clinical management could help to explain the differences in patient characteristics and outcomes observed between the peak and plateau periods of the first wave of COVID-19. In Portugal, the COVID-19 patient ICU admission peak took place between the 10^th^ and 16th weeks of 2020, while a state of national emergency was declared between weeks 12 and 14 (March 19th and April 2nd) due to the high community infection rate, effectively preventing a shortage of hospital beds and health care professionals. The nationwide number of confirmed SARS-CoV-2 infections and ICU admissions both peaked during week 13, implying that the national lockdown effectively contained the spread of the disease and reduced the number of severe COVID-19 patients and the demand on the health care system.

### Limitations

Our study had some limitations. The absence of data about the structural capacity of ICUs and hospitals throughout the study period prevented us from asserting whether the capacity of care was effectively surpassed. Even so, study centers reported no cases of mechanically ventilated patients outside the ICU. Study protocol restrictions precluded comparison between centers, and although there was a clear difference between admission rates across centers (Table 1S - Supplementary material), no minimum patient number was defined to include all centers willing to collaborate. We excluded patients with incomplete hospital outcomes to obtain a complete picture of our cohort and avoided patient groups that were still in the ICU or in the hospital with missing outcome data, as seen in earlier publications. This may have introduced a selection bias in our results. Therefore, we followed these patients a posteriori, and the overall hospital mortality rate was low (5 out of 48), without significantly affecting our results.

This was an ambispective study with relevant missing data for some variables characterizing patient severity, such as the report of criteria for ARDS or the use of some drugs, such as antibiotics. We have addressed this statistically, but it is still a relevant limitation. Finally, our study did not aim to evaluate whether specific therapies were beneficial or not, so care must be taken when interpreting and comparing our results with the literature.

## CONCLUSION

During the first COVID-19 wave, patient characteristics and clinical management in intensive care changed between peak and plateau periods. During the peak period, there was a higher rate of invasive mechanical ventilation, prone positioning, vasopressors, hydroxychloroquine and lopinavir/ritonavir. Patients in the plateau period had more comorbidities, received greater respiratory support with high flow nasal cannula, remdesivir and corticosteroid therapy and had a shorter intensive care unit length of stay. The mortality rate was similar in both periods. This study adds to the understanding of COVID-19 pandemic dynamics, contributes to health care policies and patient care and establishes a framework for future research.
