# Supplementary material for: What changed between the peak and plateau periods of the first COVID-19 pandemic wave? A multicentric Portuguese cohort study in intensive care
Source: Rev Bras Ter Intensiva. 2022 Oct-Dec;34(4):433–42. doi: 10.5935/0103-507X.20210037-en (PMC9987006; doi:10.5935/0103-507X.20210037-en)
Supplement: Supplementary file 1 [file rbti-34-04-0433-suppl1.pdf]

What changed between the peak and plateau periods of the first COVID-19 pandemic wave? A multicentric Portuguese cohort study in intensive care

O que mudou entre os períodos de pico e de platô durante a primeira onda do SARS-CoV-2? Estudo multicêntrico português em unidades de cuidados intensivos

Rui Antunes Pereira[1], Marta Sousa[2], José Pedro Cidade[3], Luís Melo[4], Diogo Lopes[1], Sara Ventura[5], Irene Aragão[6], Raul Miguel de Freitas Lima Neto[7], Elena Molinos[8], Ana Marques[9], Nelson Cardoso[10], Flávio Marino[11], Filipa Brás Monteiro[12], Ana Pinho Oliveira[13], Rogério C. Silva[14], André Miguel Neto Real[15], Bruno Sarmento Banheiro[16], Renato Reis[2], Maria Adão-Serrano[2], Ana Cracium[2], Ana Valadas[2], João Miguel Ribeiro[2], Pedro Póvoa[3], Camila Tapadinhas[3], Vítor Mendes[3], Luís Coelho[3], Raquel Maia[4], Paulo Telles Freitas[4], Isabel Amorim Ferreira[4], Tiago Ramires[4], Luís Silva Val-Flores[1], Mariana Cascão[1], Rita Alves[1], Simão C. Rodeia[5], Cleide Barrigoto[5], Rosa Cardiga[5], Maria João Ferreira da Silva[6], Bruno Vale[6], Tatiana Fonseca[7], Ana Lúcia Rios[7], João Camões[8], Danay Pérez[8], Susana Cabral[9], Maria Inês Ribeiro[10], João João Mendes[4,17], João Gouveia[2,17], Susana Mendes Fernandes[2] for the ICUCOVID19_PT Study Group[18] by the *Sociedade Portuguesa de Cuidados Intensivos*

Collaborators from the ICUCOVID19_PT study group by the *Sociedade Portuguesa de Cuidados Intensivos*

Centro Hospitalar Universitário Lisboa Norte: Susana Mendes Fernandes, Marta Sousa, Renato Reis, Maria Adão-Serrano, Ana Cracium, Ana Valadas, João Valente, Fábio Rato, Nuno Gaibino, Ria Lakhani, Dulce Correia, Inês Neves, João Miguel Ribeiro; Francisco Abecasis (Pedriatric ICU). *Hospital São Francisco Xavier, Centro Hospitalar Lisboa Ocidental:* José Pedro Cidade, Pedro Póvoa, Camila Tapadinhas, Vitor Mendes, Luís Coelho, David Nora, Maria Carolina Paulino, Antonio Tralhao, Rui Morais, Pedro Fidalgo, Patricia Moniz, Rita Santos, Vasco Costa, Luis Maia Morais, Juvenal Morais, Ivo Castro. *Hospital Fernando Fonseca:* Luis Melo, Ana Raquel Maia, Paulo Telles Freitas, Isabel Amorim Ferreira, Tiago Ramires, Nuno Martins, Mónica Anselmo, Priscila Diaz, Lisete Nunes, Raquel Silva, Liliana Antunes, Isabel Serra. *Hospital de Curry Cabral Centro Hospitalar Universitário Lisboa Central:* Rui Pereira, Diogo Lopes, Luís Val-Flores, Mariana Cascão, Rita Alves, João Teixeira, Ana Martins, Filipe Sousa Cardoso, Jorge Pelicano Paulos, Carla Maravilha, André Roberto, Filipa Cardoso, António Mesquita, Claudina Cruz, Hugo Inácio, Diogo Borges, João Crisóstomo, Catarina Pires, Joana Ferrão, Mário Ferraz, Pedro Xavier, Maria Amaral, César Vieira, Tiago Duarte, Nuno Germano. *Centro Hospital de São José, Hospitalar Universitário Lisboa Central:* Sara Ventura, Simão Rodeia, Cleide Barrigoto, Rosa Cardiga, Lúcia Proença, João Oliveira, Marta Torre, Filipa Marujo, Joana Martins, Luís Bento. *Hospital de Santo António. Centro Hospitalar Universitário do Porto:* Irene Aragão, Maria João Ferreira da Silva, Bruno Vale, Patricia Campos, Rita Pereira. *Centro Hospitalar de Vila Nova de Gaia/Espinho:* Raul Neto, Tatiana Fonseca, Ana Lúcia, Diana Adrião. *Hospital Pedro Hispano:* Elena Molinos, João Camões, Danay Pérez. *Centro Hospitalar e Universitário de Coimbra:* Ana Marques, Susana Cabral, Catarina Silva, Ana Catarino, João Francisco, João Alves; Andrea Dias (Pedriatric ICU). *Hospital do Espírito Santo de Évora:* Nelson Cardoso, Maria Inês Ribeiro, Ana Sousa, Silvia Lourenço, Manuel Chantre Lima. *Hospital Vila Franca de Xira:* Flávio Marino. *Hospital Egas Moniz, Centro Hospitalar Lisboa Ocidental:* Filipa Brás Monteiro, Pedro Santos, Francisco Coelho, João Torres, Marta Rebelo, Gabriela Almeida, Tomás Lamas, Isabel Gaspar, Isabel Simões, Eduarda Carmo. *Centro Hospitalar Tondela-Viseu:* Ana Pinho Oliveira, Carla Eira, Luís Patrão, Carla Rebelo. *Hospital Santa Luzia:* Rogério Corga da Silva. *Hospital de Abrantes, Centro Hospitalar Médio Tejo:* André Miguel Neto Real, Rui Assis, João Cardoso, David Ferreira, Nuno Catorze. *Hospital de Portimão, Centro Hospitalar Universitário do Algarve:* Bruno Sarmento Banheiro. *Hospital Dona Estefânia, Centro Hospitalar Universitário Lisboa Central: Filipa* Marujo, Joana Martins (Pedriatric ICU). *Centro Hospitalar Universitário de São João:* Carolina Batista (Pedriatric ICU).

Table 1S - Collaborating sites and included/excluded adult patients

Site	n (%)	Excluded (n) (no pneumonia/ no outcome)
Hospital de Santa Maria, Centro Hospitalar Universitário Lisboa Norte	92 (17.0)	(0/5)
Hospital São Francisco Xavier Centro Hospitalar Universitário Lisboa Ocidental	74 (13.7)	(0/4)
Hospital Professor Doutor Fernando Fonseca	63 (11.6)	(0/5)
Hospital de Curry Cabral, Centro Hospitalar Universitário Lisboa Central	58 (10.7)	(2/10)
Hospital de São José, Centro Hospitalar Universitário Lisboa Central	48 (8.9)	(0/5)
Centro Hospitalar do Porto	37 (6.8)	(2/0)
Hospital Vila Nova de Gaia - Espinho	37 (6.8)	(0/0)
Unidade Local de Saúde de Matosinhos	35 (6.5)	(0/6)
Centro Hospitalar Universitário de Coimbra	21 (3.9)	(0/2)
Hospital Espírito Santo Évora EPE	16 (3.0)	(0/3)
Hospital Vila Franca de Xira	14 (2.6)	(0/1)
Hospital de Egas Moniz, Centro Hospitalar Universitário Lisboa Oriental	11 (2.0)	(0/1)
Centro Hospitalar de Tondela-Viseu	11 (2.0)	(0/0)
Hospital de Santa Luzia, Unidade Local de Saúde Alto Minho	11 (2.0)	(3/2)
Hospital de Abrantes, Centro Hospitalar do Médio Tejo	10 (1.8)	(0/2)
Hospital de Portimão Centro Hospitalar Universitário do Algarve	3 (0.6)	(0/2)
Total	541 (100)	(7/48)

Table 2S - Clinical characteristics of children with COVID-19 in intensive care

Clinical characteristics (n = 7)	
Age (years)	6 [0 - 10]
Male gender	4 (57.1)
Comorbidities	4 (57.1)
Clinical features	
Fever	4 (57.1)
Mucocutaneous involvement	3 (42.9)
Gastrointestinal symptoms	5 (71.4)
Conjunctivitis	1 (14.3)
Lymphadenopathy	1 (14.3)
Respiratory symptoms	5 (71.4)
Heart failure, excluding shock	5 (71.4)
Neurologic symptoms	1 (14.3)
Shock at admission	3 (42.9)
Diagnosis	
MIS-C	3 (42.9)
Myocarditis	2 (28.6)
ARDS	2 (28.6)
Length of stay in pediatric ICU	9 [5 - 16]
Overall mortality	1 (14.3)

MIS-C - multisystem inflammatory syndrome in children; ARDS - acute respiratory distress syndrome; ICU - intensive care unit; IQR - interquartile range. Pediatric intensive care units from four centers reported 7 critically ill children admitted due to COVID-19 during the study period. A single death occurred in a child with severe congenital heart disease. Results expressed as median [interquartile range] or n (%).

Table 3S - Symptoms of COVID-19 in critically ill patients

Symptoms (n = 415)	n (%)
History of fever	326 (78.6)
Fatigue/Malaise	272 (65.5)
Dyspnea	271 (65.3)
Cough	253 (61)
Myalgia	153 (36.9)
Anorexia	104 (25.1)
Diarrhea	77 (18.6)
Headache	74 (17.8)
Chest pain	60 (14.5)
Vomiting/Nausea	55 (13.3)
Altered consciousness	46 (11.1)
Sore throat	45 (10.8)
Inability to walk	36 (8.7)
Rhinorrhea	32 (7.7)
Lower chest wall indrawing	30 (7.2)
Abdominal pain	26 (6.3)
Wheezing	22 (5.3)
Ageusia	21 (5.1)
Anosmia	20 (4.8)
Others	24 (5.8)
